# Leadership training programs in graduate medical education: a systematic review

**DOI:** 10.1186/s12909-020-02089-2

**Published:** 2020-06-02

**Authors:** Bharat Kumar, Melissa L. Swee, Manish Suneja

**Affiliations:** 1 Internal Medicine in the Division of Immunology at the University of Iowa Hospitals and Clinics, 200 Hawkins Drive, Iowa City, IA 52245 USA; 2 Internal Medicine in the Division of Nephrology at the University of Iowa Hospitals and Clinics, Iowa City, IA USA; 3 Medicine Residency Program Director in the Department of Internal Medicine, University of Iowa Hospitals and Clinics, Iowa City, IA USA

**Keywords:** Leadership, Graduate medical education, Professionalism

## Abstract

**Background:**

With the increasing recognition that leadership skills can be acquired, there is a heightened focus on incorporating leadership training as a part of graduate medical education. However, there is considerable lack of agreement regarding how to facilitate acquisition of these skills to resident, chief resident, and fellow physicians.

**Methods:**

Articles were identified through a search of Ovid MEDLINE, EMBASE, CINAHL, ERIC, PsycNet, Cochrane Systemic Reviews, and Cochrane Central Register of Controlled Trials from 1948 to 2019. Additional sources were identified through contacting authors and scanning references. We included articles that described and evaluated leadership training programs in the United States and Canada. Methodological quality was assessed via the MERSQI (Medical Education Research Study Quality Instrument).

**Results:**

Fifteen studies, which collectively included 639 residents, chief residents, and fellows, met the eligibility criteria. The format, content, and duration of these programs varied considerably. The majority focused on conflict management, interpersonal skills, and stress management. Twelve were prospective case series and three were retrospective. Seven used pre- and post-test surveys, while seven used course evaluations. Only three had follow-up evaluations after 6 months to 1 year. MERSQI scores ranged from 6 to 9.

**Conclusions:**

Despite interest in incorporating structured leadership training into graduate medical education curricula, there is a lack of methodologically rigorous studies evaluating its effectiveness. High-quality well-designed studies, focusing particularly on the validity of content, internal structure, and relationship to other variables, are required in order to determine if these programs have a lasting effect on the acquisition of leadership skills.

## Background

The American healthcare system is in a state of tremendous flux, with the role of physicians and other healthcare providers rapidly changing to keep up with technological advances, financial restructuring, and the adoption of new societal and technological standards. Leadership training has been proposed as a means of managing these changes and ensuring that physicians are able to navigate their changing roles as health providers [1]. This follows the example of the business community, where leadership development is considered a high priority among managers.

Leadership is a term that is used to describe the ability of an individual to guide an organization or group of individuals [2]. In contrast to managers, leaders tend to exert authority through words and actions to convince followers towards a fulfillment of a vision, rather than through reward or punishment to induce control over subordinates. While considerable controversy exists over what styles and skills are necessary for effective leadership, this has become a burgeoning field of study. Sitkin and colleagues have identified six interrelated domains of leadership, namely personal, relational, contextual, inspirational, supportive, and responsible [3]. Each domain of leadership has an associated conceptual basis, group of behaviors, and set of skills. While an in-depth discussion of these domains and their application to medical education is beyond the scope of this article, acknowledgement of the importance of leadership and the heterogeneity of leadership styles is essential towards understandings the role of leadership development in graduate medical education.

With regards to medicine, it has been noted by several authors that there is a lack of leadership training for physicians in academic medical centers [4]. There has been a tendency to believe that leadership skills are acquired on the job and cannot be taught effectively, leaving a deficit of highly qualified physician leaders [5]. However, it is being increasingly recognized at least some leadership skills can be cultivated through formal and informal education, and that effective leaders cultivate their leadership capacity through diligent practice [6, 7]. Despite these calls for leadership training, the Accreditation Council on Graduate Medical Education (ACGME) has not yet articulated a specific position on leadership training. Currently, leadership skills constitute sub-competencies in two of the six competencies promoted by the Outcome Project of the ACGME, namely (1) professionalism and (2) interpersonal skills and communication skills [8]. New program requirements proposed by the ACGME have focused on the medical team, which is intimately linked to team leadership. Further, some of the members of the Council of Review Committee Residents Leadership Subcommittee of the ACGME have supported the need for leadership training, although they state that their opinions are not necessarily the official position of the ACGME [9].

There are scarce data about how leadership training programs are implemented in the framework of graduate medical education, i.e. residency and fellowship programs. Even less is known about the impact of these programs. Two systematic reviews have been performed regarding physician leadership training programs, but these did not specifically focus on resident and fellow physicians in North America, who operate in a very distinct environment and face unique challenges compared to physician executives, faculty members, and trainees in other geographic settings [10, 11]. In addition, a third systematic review focused on resident and fellow physicians in North America but did not assess the methodological rigor of included studies [12]. In order to document and characterize the impacts of these programs on leadership development, as well as provide direction for how to frame medical education interventions to study leadership development in graduate medical education, we have conducted a systematic review of literature.

## Methods

**Fig. 1:**
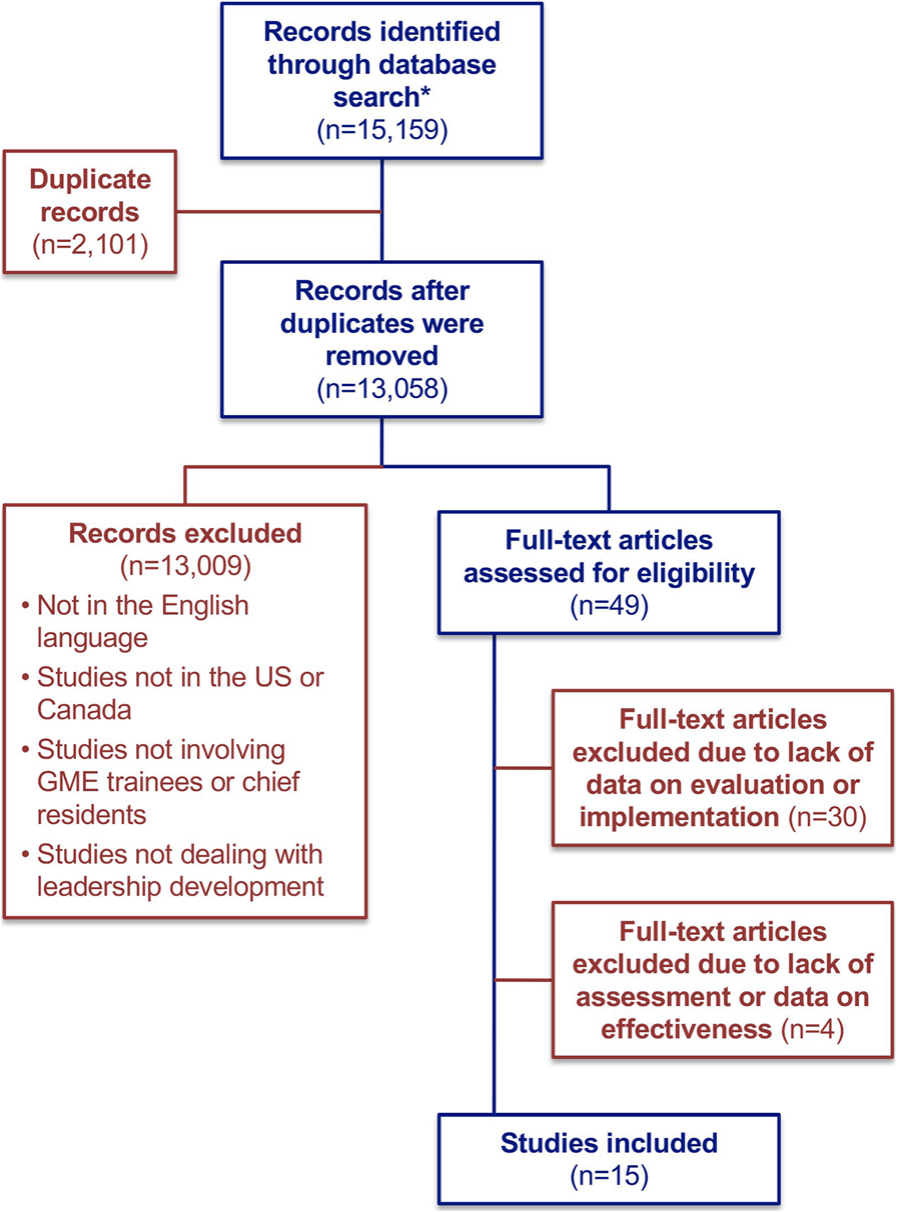
Literature Search Strategy. Legend: MEDLINE: 12,851 citations; CINAHL: 586; EMBASE: 301; Cochrane Systematic Reviews: 0; Cochrane Central Register of Controlled Trials: 0; PsychNet: 0; ERIC: 0

The Preferred Reporting Items for Systematic Reviews and Meta-Analyses (PRISMA) statement was used to guide our literature search and report (Supplement). Figure 1 documents how we selected articles for inclusion in our analysis, and Table 1 documents the characteristics of the included studies.

**Table 1:** Characteristics of Included Studies

**Study**	**Study Design**	**Setting**	**Sample Size**	**Age**	**Sex**	**Specialty**	**Level of Training**	**Description of Program**	**Topics Covered**	**Follow-up Period**	**Outcome Metrics**
Whitman, 1988 [13]	Prospective case series	United States (participants from 138 residency programs)	180	Not reported	Not reported	21 specialties, including Internal Medicine, Surgery, and Family Medicine	Chief Resident	Two-day workshop presenting information on a management skill in 45 min followed by 60-min small group exercise on applying the skills	Time management Delegation of work Feedback Motivation Stress management Conflict resolution Clinical teaching Team building	None	Survey of each lecture and small-group exercise (5-point scale)
Doughty et al., 1991 [14]	Retrospective case series	United States (participants from 50 institutions)	117	Not reported	Not reported	Pediatrics	Chief Resident	3-day experiential workshop	Leadership roles Group functioning Personal leadership skills and styles Dealing with conflict Effective feedback Stress management Working with hospital administration	6 months	Evaluation of activities (10-point Likert Scale)
Mygdal et al., 1991 [15]	Prospective case series	Texas Family Practice Chief Resident Leadership Conference	27	Not reported	Not reported	Family Medicine	Chief Resident	Workshops (stress coping skills, leadership skills), Small group discussions (supplementing the workshops), Plenary speeches (three talks by leaders in Texas Family Medicine), Concluding planning session	Leadership skills Stress management	1 year	Self-rating scale, subdivided into two subscales: Stress Management and Leadership Skills; Reactions to conference scale, Conference Evaluation Scale
Evans et al., 1997 [16]	Prospective case control	University of Washington Family Practice Residency Network	78 (64 control, 14 study group)	28.7	42.3% Male	Family Medicine	PGY-1	A series of outdoor activities led by professional facilitators	Group dynamics Trust-building Problem-solving processes Communication patterns	None	Self-assessment survey instrument: 7-point Likert Scale
Crites and Schuster, 2004 [17]	Prospective case series	Wright University	12	Not reported	Not reported	Internal Medicine/ Pediatrics	PGY-1 to PGY-4	Monthly 30-min seminars	Basic coding Revenue management Optimizing coding to enhance reimbursement Physician personal finance Insurance systems and payment mechanisms Dynamics of group practice Getting a good job Accounts receivable management Accounts payable management Human resources Risk management Regulatory restrictions in practice	None	Self-assessment (Likert scale from 1 to 5); Curriculum Evaluation (Likert scale from 1 to 5)
Lee, Tse and Naguwa, 2004 [18]	Prospective case series	University of Hawaii	10	Not reported	Not reported	Pediatrics	PGY-2	3-h interactive workshop	Leadership skills	None	Self-assessment (Likert scale from 1 to 5)
Stoller, et al., 2004 [19]	Prospective case series	University of Washington	32	27.7	25% Female	Internal Medicine	PGY-1, PGY-2	Three 2-h sessions	Managing a ward team and leadership skills Resident role as a teacher Microskills of teaching and problems faced by residents	None	Self-assessment (Likert scale from 1 to 5)
Hemer et al., 2007 [20]	Prospective case series	Mayo Clinic	16	Not reported	Not reported	Pathology	Senior Residents	Annual course consisting of 6 sessions, each of which were 1–2 days long (average 10 h), followed by a capstone seminar	Leadership and management Managing change and interpersonal skills Personnel issues and quality improvement Informatics Finance	None	Course Evaluation: Pre- and Post-test evaluation
Stergiopoulos et al., 2009 [21]	Prospective case series	University of Toronto	52	Not reported	Not reported	Psychiatry	PGY-2 & PGY-4	Four workshops, utilizing case studies, “think-pair-share”, and “buzz groups”	Teamwork Conflict resolution and negotiation Quality improvement Program planning and evaluation Leadership skills Mental health and addiction reform Organizational structures Self and career development	None	Workshop ratings (Likert scale from 0 to 5 for each topic covered assessing if the objectives were met)
Pettit et al., 2011 [22]	Retrospective case series	University of Iowa	9	Not reported	Not reported	Neuro-surgery	PGY-1 to PGY-5	Monthly 1-h workshops	Leadership styles Conflict management Communication styles Motivation	1 year	20-item retrospective pre-test and post-test
Kuo et al., 2010 [23]	Retrospective case series	University of California San Francisco	24	Not reported	Not reported	Pediatrics	PGY-1 to PGY-3	3-year residency program. Completion of a collaborative child advocacy project (protected time: one-half day a week during non-seminar outpatient and elective rotation), structured mentoring	Leadership skills Advocacy	None	Awards and Grants obtained by graduates
Brandon and Mullan, 2013 [24]	Prospective case series	University of Michigan	44	Not reported	Not reported	Radiology	Resident and Fellow Physicians	Seven modules of 90-min sessions	Costing analysis Quality improvement Profiles of practice groups Group governance Data collection and process improvement Healthcare economics Negotiation and conflict management	None	Pre- and post-test assessments
Cole, et al., 2017 [25]	Prospective case series	University of Florida	10	Not reported	Not reported	Anesthesiology	Senior residents (PGY4)	2-week rotation in the Operating Room consisting of guided readings and daily debriefings	Communication Teamwork Situational awareness Decision-making	None	Self-assessment and raters by others in the Operating Room
Itri, et al., 2017 [26]	Prospective Case Series	University of Virginia	21	Not reported	Not reported	Radiology	Radiology Residents	1 workshop with group discussion, role-play, and simulation	Communication Interpersonal skills	None	Self-assessment
Hill, et al., 2018 [27]	Prospective case series	Staten Island University Hospital	7	Not reported	Not reported	Surgery	Senior Residents (PGY3–5)	Small-group discussions	Leadership skills (not further defined)	None	Pre- and post-test assessments

### Search strategy

Two authors (B.K. and M.L.S.) searched MEDLINE (from 1948 to January 31th, 2019), EMBASE (from 1988 to January 31th, 2019), CINAHL (from 1994 to January 31th, 2019), Cochrane Central Register of Controlled Trials (from 1996 to January 31th, 2019), Cochrane Systematic Reviews (from 1993 to June 30th, 2017), ERIC (from 1965 to January 31th, 2019) and PsycNet (1970 to January 31th, 2019), for potentially relevant studies. These searches were limited to articles written in English. A Boolean search strategy using a series of three terms was employed in order to obtain these articles. Search terms included (“medical education,” OR “residency,” OR “fellowship,” OR “medical training”) AND (“leadership” OR “management” OR “advocacy”) AND (“development” OR “skills” OR “training” OR “workshop” OR “session” OR “curriculum” OR “activities” OR “syllabus” OR “modules”).

Additionally, we supplemented this search by scanning the references of identified studies, as well as the related three systematic reviews [10–12]. In order to address publication bias, we also attempted to contact the authors of studies that were ultimately included in our review. The contact information of six authors was obtained, but only one had replied back with four articles, but none of these were new, previously unidentified studies.

### Eligibility criteria

All qualitative and quantitative studies written in the English language that contained data regarding the implementation and evaluation of leadership training programs during graduate medical education were eligible. These graduate medical education programs consisted of residency and fellowship programs in either Canada or the United States. For our purposes, we included chief residents, who, depending on the training program, may be senior resident physicians or very recent graduates of residency programs. Due to the similarities in the structure of medical education training programs between Canada and the United States, we decided to include both countries.

We excluded studies that did not specifically deal with leadership training in graduate medical education, such as studies solely describing practice management or quality improvement. Similarly, we excluded studies that were not designed towards trainees in graduate medical education.

### Study selection process

The two authors independently screened titles and abstracts compiled during the literature search. Full text of relevant articles was obtained based on the eligibility criteria noted above. Abstracts without concomitant full studies were excluded as they would be unlikely to provide enough detailed information for the systematic review. Conflicts were resolved by discussion and an apparatus was set up for a third author (M.S.) to resolve any discrepancies.

### Data abstraction

The two authors jointly developed criteria for data abstraction. These included study design, physician characteristics, and outcomes. We discussed any studies that were subject to conflict, and calculated the kappa statistic. The MERSQI (Medical Education Research Study Quality Instrument) criteria were used to appraise the methodological quality of included studies (Table 2).

**Table 2:** Methodological Quality of Included Studies

**Study**	**Study Design**^**a**^	**Sampling:** **Institutions**^**b**^	**Sampling:** **Response Rate**^**c**^	**Type of Data**^**d**^	**Validity: Internal structure**^**e**^	**Validity: Content**^**f**^	**Validity: Relationship to other variables**^**g**^	**Data analysis:** **Sophistication**^**h**^	**Data analysis:** **Appropriate**^**i**^	**Outcome**^**j**^	**MERSQI Score**
Whitman, 1988 [13]	1	1.5	1.5	1	0	0	0	1	1	1.5	8.5
Doughty et al., 1991 [14]	1	1.5	0.5	1	0	0	0	1	1	1	7
Mygdal et al., 1991 [15]	1	0.5	0.5	1	0	0	0	1	1	1	6
Evans et al., 1997 [16]	1	1.5	0.5	1	0	0	0	1	1	1	7
Lee, Tse and Naguwa, 2004 [18]	1.5	0.5	1.5	1	1	0	0	2	1	1	7.5
Stoller, et al., 2004 [19]	1	1	1	1	0	0	0	1	1	1	7
Crites and Schuster, 2004 [17]	1	0.5	0.5	1	0	0	0	1	1	1	6
Hemer et al., 2007 [20]	1	0.5	0.5	1	0	0	0	1	1	1	6
Stergiopoulos et al., 2009 [21]	1	0.5	0.5	1	0	0	0	1	1	1	6
Kuo et al., 2010 [23]	1	0.5	1.5	3	0	0	0	1	1	1	9
Pettit et al., 2011 [22]	2	0.5	0.5	1	0	0	0	2	1	1	8
Brandon and Mullan, 2013 [24]	1	0.5	0.5	1	0	0	0	2	1	1	7
Cole, et al., 2017 [25]	1	0.5	1.5	1	0	0	0	1	1	1	7
Itri, et al., 2017 [26]	1	0.5	1.5	1	0	0	0	1	1	1	7
Hill, et al., 2018 [27]	1	0.5	1.5	1	0	0	0	1	1	1	7

Legend: The MERSQI is a validated tool to appraise the quality of medical education research. The maximum score is 18, and is calculated as follows:

a. Study design: Single group cross-sectional (1); single group pre- and post-test (1.5); nonrandomized, 2 group (2); randomized controlled experiment (3)

b. Sampling: Single institution (0.5); 2 institutions (1); More than 2 institutions (1.5)

c. Response rate: Not applicable (0); Response rate < 50% or not reported (0.5); Response rate 50–74% (1); Response rate > 75% (1.5)

d. Type of data: Assessment by study subject (1); Objective measurement (3)

e. Internal structure: Not reported/NA (0); Reported (1)

f. Content: Not reported/NA (0); Reported (1)

g. Relationships to other variables: Not reported/NA (0); Reported (1)

h. Sophistication of analysis: Descriptive analysis only (1); Beyond descriptive analysis (2)

i. Appropriateness of analysis: Inappropriate (0); Inappropriate (1)

j. Outcome: Satisfaction, attitudes, perceptions (1); Knowledge, skills (1.5); Behaviors (2); Patient/health care outcome (3)

### Synthesis of included studies

A narrative review was drafted based on the included studies. While the original intent was to perform a meta-analysis, this could not be performed due to the heterogeneity of study designs and outcomes and absence of an adequate sample size to test different variables.

## Results

### Literature search

Fifteen thousand one hundred fifty-nine citations were obtained through the literature search strategy, of which 46 articles were deemed potentially relevant. Thirty of these did not include data on either evaluation or implementation, resulting in 15 unique studies [13–31]. Among these, four were excluded since they were descriptive of new leadership curricula but lacked information on how effectiveness was assessed [28–31]. The kappa statistic was calculated and was 0.93 for the 15 included studies; the two authors disagreed about the inclusion of only one article which was adjudicated and it was determined that it should be included.

### Study and population characteristics

Fifteen studies were altogether included in the analysis. Of the 15 unique studies identified, 12 were prospective case series and three were retrospective case series. Fourteen were quantitative in nature and one had a qualitative component. Surveys were used to determine the effect of the intervention in 12 of the studies, while another looked at outcomes in terms of awards, grants, and projects either won or executed by participants after completion. Among those evaluated by survey instruments, eight used self-assessment surveys and seven used course evaluations. Seven used pre- and post-test surveys while an eighth used a post-test and retrospective pre-test. Geographically, Fourteen studies were conducted in the United States and one was conducted in Canada. Two of these involved participants from multiple institutions [13, 15].

Altogether, there were 639 residents or fellows participating in the 15 studies. All participants were graduate medical education trainees, but these varied among different residency and fellowship training programs. Three were designed specifically for chief residents and three for senior residents, while the rest of the nine were open to residents of all different training years. Only one explicitly included fellow physicians in addition to resident physicians. Details regarding age and gender were not available for 13 of the 15 studies.

There was considerable variety in content, methods of instruction and the duration of intervention. Four studies did not enumerate the specific topics beyond development of “leadership skills.” The most common topics listed included teamwork, communication, and conflict resolution, which were seen in seven of the studies, followed by stress management in 4 studies. Ancillary topics in advocacy, personal finance, quality improvement, public health, and business management were also seen in several studies. A full list of topics covered is noted in Table 1.

The methods of instruction also varied: nine were workshops, four were didactic sessions, one was a series of small-group discussions, and one was an entire three-year residency program. Even among these, there was considerable heterogeneity in the length of time of each workshop/seminar, with some sessions as short as 30 min and others lasting for over 90 min. Additionally, the duration of training ranged considerably from a one-day workshop to a three-year residency program.

### Quality assessment

Only two of the 15 studies included details on the age or gender of the participants, and so the representativeness of these studies compared to the general population of GME trainees is unclear. The 12 prospective case series did not detail specifically about how participants were selected, aside from being members of the residency or fellowship program. Similarly, no exclusion criteria were elaborated.

Self-reported questionnaires were utilized in 14 of the 15 of the studies. However, only one was noted to be validated by authors. Also, none described blinding of outcomes assessment. One study reported follow-up after 6 months; two additional studies reported follow-up after 12 months. The remaining 12 did not have follow-up. One study looked at the outcomes in terms of awards and grants 6 years after graduation of the first set of cohorts.

MERSQI scores were calculated for each of the included studies, and varied from 6 to 9 out of a possible maximal score of 18 (Table 2).

### Impact of leadership programs

Among the seven studies that used pre- and post- self-assessment surveys, all showed improvement in the perceptions and attitudes of knowledge and leadership skills, although measured in different ways. In the six surveys evaluating the programs themselves, there was broad satisfaction at the quality and content of the program.

## Discussion

To our knowledge, this systematic review is the first to characterize and appraise leadership development programs specifically among graduate medical education trainees. Of note, one prior systematic review had appraised the strength of conclusions using the Best Evidence in Medical Education (BEME) Index, but did not appraise the methodology, framework, and results in total. To do so, we employed the MERSQI. The MERSQI is a validated and widely used instrument to assess quality of educational interventions, and, among the most commonly used instruments (BEME, MERSQI, modified Newcastle-Ottawa Scale [m-NOS]), it is most strongly associated with study quality, as assessed by the Strengthening the Reporting of Observational Studies in Epidemiology (STROBE) statement [32]. By using the MERSQI to more critically inspect these studies, our analysis informs educators about how to build upon what has been previously published to better structure leadership development programs in graduate medical education training programs.

The biggest limitation in the design of these studies is the lack of validity (Internal structure, content, and relationship to other variables). Only one article (Lee, Tse, and Naguwa [18]) documented efforts to ensure that there was validity in the internal structure of their intervention. None sought to validate content and relationship to other variables. We strongly recommend that future studies critically examine and report the steps that they take to ensure validity. Admittedly, this is difficult given the absence of a single definition of leadership and the tendency for leadership to be viewed as a situationally- and contextually-dependent competency [3–5]. However, it should not deter investigators in exploring and analyzing how the variables being measured may link to the concept of leadership.

Likewise, future studies need to examine outcomes on patient/healthcare and behaviors. Only one of the 15 examined knowledge or skills (Whitman [13]) while the others studied satisfaction, attitudes, and perceptions as outcomes. Because leadership is so intrinsically tied to behavioral patterns, evaluation of these sorts of outcomes is essential [1, 2]. Likewise, leadership is consistently mentioned in the articles included in our analysis as potentially transformative for healthcare, yet the impacts of these interventions on such outcomes are not measured or documented. This is an understandable limitation given the practical challenges of designing medical education studies but it is difficult to interpret the significance of these studies without data regarding more meaningful outcomes that are more closely tied to leadership.

Of course, our analysis has some important limitations. First, because leadership encompasses several overlapping concepts, the foci of these studies were slightly different. Some articles did not break down what types of leadership skills were emphasized in these training programs, while others provided significantly more detail. This variability in content and focus underline the importance of looking critically at leadership as a set of overlapping competencies. Moreover, it reinforces the need to scrutinize study design and methodology of prior published studies, over specific results, since it is unclear how much overlap there is in content between the curricula of the 15 included studies.

Second, the outcomes reported were largely self-reported through non-validated questionnaires. Except for Kuo’s report of the establishment of a three-year residency program, all of the included studies used either pre- and post-test knowledge-based assessments, or self-assessments. Six of the studies that evaluated the course content and composition demonstrated that participants were satisfied, according to the authors’ conclusions. Additionally, 6 studies demonstrated there was a positive impact on their own perception that they had learned about leadership skills. While these are helpful in determining what was learned and how learners viewed their experiences, it does not necessarily provide information about how leadership training impacts behaviors or institutional culture. The absence of follow-up beyond the initial training course in all but three studies also makes it difficult to determine what lasting impact these training programs had on participants.

Thirdly, inclusion and exclusion criteria were not clearly elucidated in the included studies. In the absence of this information, it is difficult to ascertain selection bias or drop-out between training sessions. Similarly, demographic information regarding age and gender were missing in all but one study. These findings preclude generalization of any particular conclusion about leadership training in graduate medical education.

Our systematic review does have certain methodological shortcomings. We limited our search to articles focusing on leadership, but due to the ambiguities regarding the precise definition of leadership, we may have missed articles related to “team leaders,” “managers,” “self-management” or other topics within the realms of leadership training. It is therefore vital to establish clearer definitions of leadership in the context of healthcare and to articulate what competencies define physician leadership. Using clearer definitions of leadership may facilitate investigators to better describe their efforts to uphold the validity of contents, internal structure, and relationship to other variables.

Strengths of our systematic review include the use of multiple databases and the solicitation of other references by both searching the reference lists and by attempting to contact authors of the published material. The methodological rigor of the review was upheld through strict adherence to the PRISMA statement, and each study was evaluated by the MERSQI, a validated instrument to appraise the methodological quality of studies.

## Conclusion

This systematic analysis has identified a significant absence in the publication of rigorously designed and evaluated leadership training programs. There is particularly a lack of studies that describe the validity of content, relationship to other variables, and internal structure. What has been published suggests that leadership training is a worthwhile endeavor, and that participants do learn more about leadership and are favorably disposed to workshops and seminars. We recommend that further high-quality research be undertaken in order to better understand how leadership skills can best be imparted for trainees in graduate medical education, and how formal training programs influence more long-term and objective measures of leadership and management.

## Supplementary information

**Additional file 1.** Appendix 1: PRISMA Checklist.

## Data Availability

All data generated or analysed during this study are included in this published article.

## Abbreviations

ACMGE: Accreditation Council for Graduate Medical Education
BEME: Best Evidence in Medical Education
CINAHL: Cumulative Index to Nursing and Allied Health Literature
EMBASE: Excerpta Medica Database
MERSQI: Medical Education Research Study Quality Instrument
m-NOS: modified Newcastle-Ottawa Scale
PRISMA: Preferred Reporting Items for Systematic Reviews and Meta-Analyses
STROBE: Strengthening the Reporting of Observational Studies in Epidemiology
