# Supplementary figures and images for: Leadership training programs in graduate medical education: a systematic review
Source: BMC Med Educ. 2020 Jun 2;20:175. doi: 10.1186/s12909-020-02089-2 (PMC7268469; doi:10.1186/s12909-020-02089-2)

**Appendix 1: PRISMA Checklist**
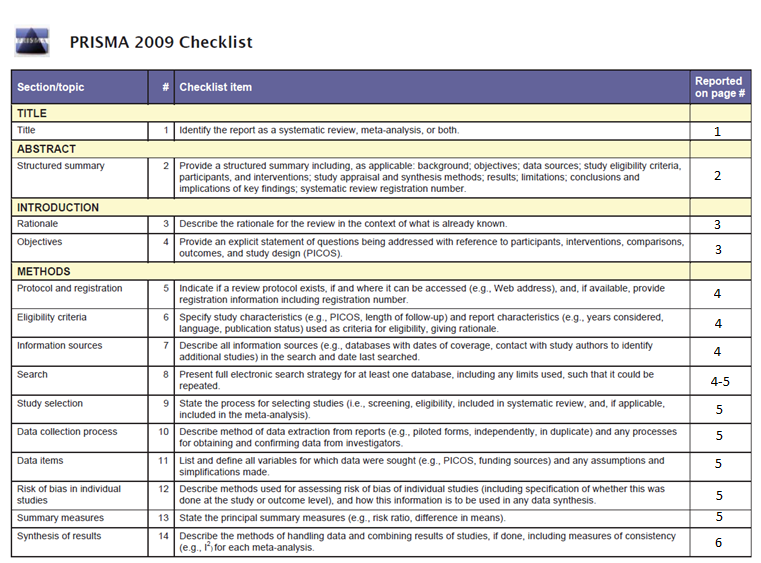

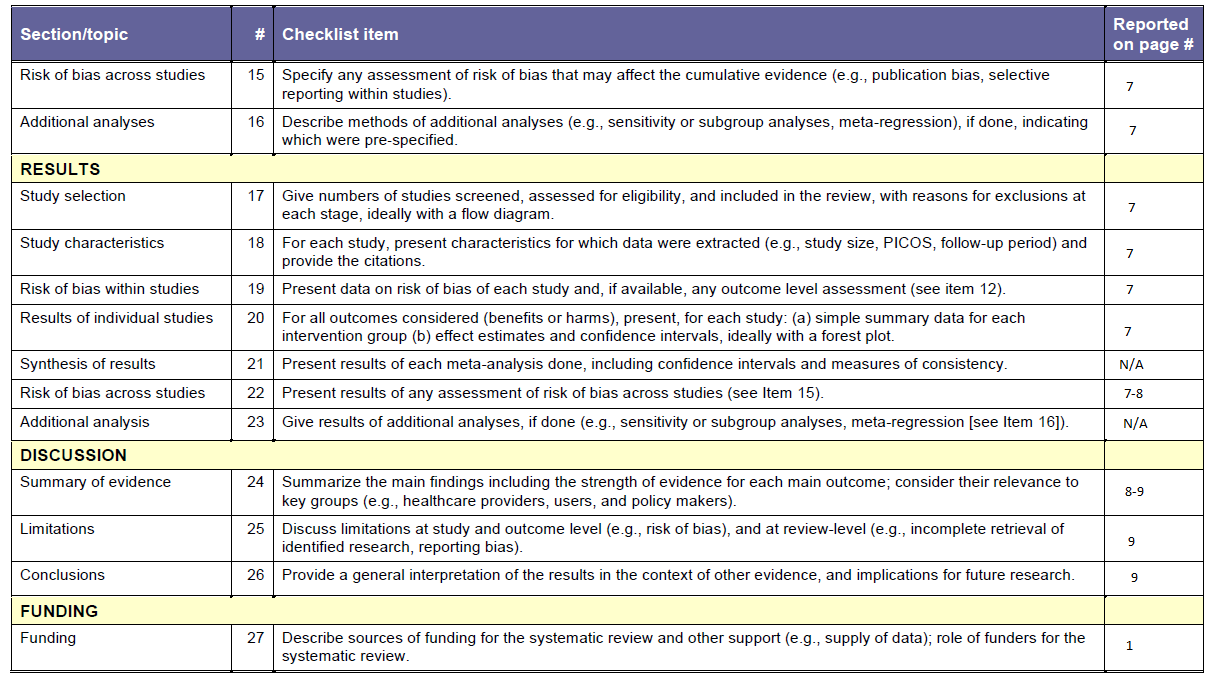

Supplement: Supplementary file 1 — Additional file 1. Appendix 1: PRISMA Checklist. [file 12909_2020_2089_MOESM1_ESM.docx]
